# Enteric neurospheres retain the capacity to assemble neural networks with motile and metamorphic gliocytes and ganglia

**DOI:** 10.1186/s13287-023-03517-y

**Published:** 2023-10-05

**Authors:** Jeng-Chang Chen, Wendy Yang, Li-Yun Tseng, Hsueh-Ling Chang

**Affiliations:** 1 Department of Surgery, Chang Gung Children’s Hospital, College of Medicine, Chang Gung University, 5, Fu-Shin Street, Kweishan, Taoyuan, 333 Taiwan; 2 Graduate Institute of Clinical Medicine, College of Medicine, National Taiwan University, Taipei, 100 Taiwan; 3 Pediatric Research Center, Chang Gung Children’s Hospital, College of Medicine, Chang Gung University, Taoyuan, 333 Taiwan

**Keywords:** Apoptosis, Enteric neurosphere, Enteric neural stem cell, Enteric neural network, Enteric nervous system, Differentiation, Neurosphere medium, Self-renewal medium

## Abstract

**Background:**

Neurosphere medium (NSM) and self-renewal medium (SRM) were widely used to isolate enteric neural stem cells (ENSCs) in the form of neurospheres. ENSCs or their neurosphere forms were neurogenic and gliogenic, but the compelling evidence for their capacity of assembling enteric neural networks remained lacking, raising the question of their aptitude for rebuilding the enteric nervous system (ENS) in ENSC therapeutics. It prompted us to explore an effective culture protocol or strategy for assembling ENS networks, which might also be employed as an in vitro model to simplify the biological complexity of ENS embedded in gut walls.

**Methods:**

NSM and SRM were examined for their capacity to generate neurospheres in mass culture of dispersed murine fetal enterocytes at serially diluted doses and assemble enteric neural networks in two- and three-dimensional cell culture systems and ex vivo on gut explants. Time-lapse microphotography was employed to capture cell activities of assembled neural networks. Neurosphere transplantation was performed via rectal submucosal injection.

**Results:**

In mass culture of dispersed enterocytes, NSM generated discrete units of neurospheres, whereas SRM promoted neural network assembly with neurospheres akin to enteric ganglia. Both were highly affected by seeding cell doses. SRM had similar ENSC mitosis-driving capacity to NSM, but was superior in driving ENSC differentiation in company with heightened ENSC apoptosis. Enteric neurospheres were motile, capable of merging together. It argued against their clonal entities. When nurtured in SRM, enteric neurospheres proved competent to assemble neural networks on two-dimensional coverslips, in three-dimensional hydrogels and on gut explants. In the course of neural network assembly from enteric neurospheres, neurite extension was preceded by migratory expansion of gliocytes. Assembled neural networks contained motile ganglia and gliocytes that constantly underwent shapeshift. Neurospheres transplanted into rectal submucosa might reconstitute myenteric plexuses of recipients’ rectum.

**Conclusion:**

Enteric neurospheres mass-produced in NSM might assemble neural networks in SRM-immersed two- or three-dimensional environments and on gut explants, and reconstitute myenteric plexuses of the colon after rectal submucosal transplantation. Our results also shed first light on the dynamic entity of ENS and open the experimental avenues to explore cellular activities of ENS and facilitate ENS demystification.

**Supplementary Information:**

The online version contains supplementary material available at 10.1186/s13287-023-03517-y.

## Introduction

In neurogastroenterology, the culture systems of enteric neural stem cells (ENSCs) were destined for the generation of spherical aggregates (neurospherogenesis) from enzymically dissociated enterocytes (referring to all the cells dissociated from intestinal tissues), or the induction of ENSC differentiation toward enteric neurons and gliocytes. They mostly took advantage of the knowledge and protocols already available in the central or peripheral nervous system (CNS or PNS) stem cell biology. However, CNS or PNS stem cell culture systems themselves were not standardized in terms of their basal medium, growth factors, hormones or other supplements [1, 2], let alone ENSC culture protocols, which varied significantly from laboratory to laboratory [3–6] and even from report to report in the same research group [7, 8]. To date, there has been no general consensus on an optimal protocol for the isolation or propagation of enteric neurospheres [3–6, 9–11], and the induction of ENSC differentiation [6, 7, 11], making it difficult to compare the results among studies and assess the neurotrophic or neuroinhibitory effects of supplemented factors.

Neurosphere medium (NSM) [4, 7, 12–16] and self-renewal medium (SRM) [3, 17–20], coined by Cooper et al. [13] and Joseph et al. [17], respectively, represented the two most popular formulas in ENSC applications. Both were supportive of enteric neurospherogenesis and even ENSC differentiation given component modifications [7, 16–18]. Paradoxically, neurogenesis and gliogenesis in modified NSM [7, 16] or SRM [17, 18] did not reportedly come with the assembly of neural networks that hallmark the highly differentiated neural cells of the enteric nervous system (ENS). In our recent study, mass culture of dispersed enterocytes might lead to neural network assembly in SRM (with or without chicken embryo extract) but not NSM [21]. It contrasted sharply with clonal culture in prior studies, where SRM-related protocols failed to support neural network development [17, 18]. This distinction raised the question of whether seeding cell doses in culture wells were crucial to in vitro assembly of enteric neural networks. As we know, ENS networks are embedded in the gut walls, precluding their exploration. Thus, the assembly of ENS networks in vitro may simplify the biological complexity of embedded ENS so as to facilitate our investigations into its neural cell activities. In this study, we evaluated the influences of seeding cell doses on neurospherogenesis and neural network assembly in NSM and SRM, recorded the courses of neurite outgrowth from neurospheres, and explored the cellular activities of assembled ENS networks. Our studies showed that NSM was ideal for neurosphere enrichment in mass culture of dispersed enterocytes, whereas SRM exhibited superiority in driving neuronal differentiation and wiring. Both were highly affected by the seeding cell dose of dispersed enterocytes. Enteric neurospheres mass produced in NSM not only retained the capacity to assemble neural networks on SRM-immersed coverslips and gut explants as well as in three-dimensional (3D) hydrogels, but might also reconstitute myenteric plexuses of the colon after transplantation. Moreover, the assembled ENS networks were the dynamic organizations, containing motile and shapeshifting ganglia and gliocytes.

## Methods

### Mice

Inbred FVB/N and transgenic FVB/NCrl-Tg(Pgk1-EGFP)01Narl mice were purchased from National Laboratory Animal Center (Taipei, Taiwan) at the age of 6–8 weeks. Animals were housed in Animal Care Facility at Chang Gung Memorial Hospital (CGMH) under the standard guidelines from "Guide for the Care and Use of Laboratory Animals" and with the approval of CGMH Committee on Animal Research. Females were caged with males in the afternoon and checked for vaginal plugs the following morning. The day of plug detection was designated as day 0 of the pregnancy.

### Harvest of intestine in fetal mice

For fetal intestine harvesting, pregnant mice were subjected to midline laparotomy under anesthesia with subcutaneous injection of zoletil (50 mg/kg) and xylazine (1 mg/kg) to expose uteri on their gestational day 14. The fetus was delivered through hysterotomy and immediately washed with saline. Following decapitation and laparotomy, fetal intestine was obtained and bathed in saline.

### Mass culture of dispersed enterocytes

Fetal murine gut was treated with 1 mg/mL Collagenase/Dispase (Roche, Mannheim, Germany) in phosphate-buffered saline (PBS) for 30–45 min at 37 °C. Digested tissue was triturated and washed. Then, dispersed enterocytes were seeded into 6-well culture plates (Tissue culture testplate 6, ∅33.9 mm, TPP 92006) flooded with 1.2 ml NSM [4, 7, 12–15] or SRM [18, 20, 22, 23] at the cell doses of 0.3–10 × 10^5^. Serum-free NSM (100 ml) was prepared on the basis of Dulbecco's Modified Eagle Medium/Nutrient Mixture F-12 (DMEM/F12, 96 ml) supplemented with epidermal growth factor (EGF, 20 ng/ml; Peprotech, London, UK), basic fibroblast growth factor (bFGF, 20 ng/ml; Peprotech), 1 ml penicillin–streptomycin, 1 ml N2 (Gibco) and 2 ml B27 (Gibco) supplements. SRM (100 ml, free from chicken embryo extract) was made up of 65 ml high-glucose DMEM, 30 ml neurobasal medium (Gibco), 100 µl retinoic acid (117 µM), 1 ml penicillin–streptomycin, 1 ml N2 supplement, 2 ml B27 supplement, 100 μl 2-mercaptoethanol (50 mM), bFGF (20 ng/ml) and insulin-like growth factor 1 (IGF-1, 20 ng/ml; Peprotech). Culture medium was replaced every 3–4 days.

### Enumeration of mitotic p75^+^ ENSCs by carboxyfluorescein diacetate succinimidyl ester (CFSE) labeling [24]

CFSE labeling was initiated by mixing 5–10 × 10^6^ dispersed enterocytes/ml DMEM with 10 µM CFSE in a volume of DMEM equal to that of enterocyte suspension. The cell labeling process was maintained at 4 °C with periodical agitation for 15 min and then quenched by adding an equal volume of 10% fetal bovine serum (Hyclone, Logan, UT) in DMEM to the sample for 1 min. The CFSE-labeled cells were washed twice, recounted and nurtured at the dose of 10^6^ cells/1.5 ml NSM or SRM in 6-well culture plates. Next day, cells were trypsinized by Accutase (STEMCELL technologies), surface-stained with anti-p75 (Clone 25–8, LSBio) followed by Alexa Fluor 647-conjugated goat anti-rat IgG (BioLegend), and finally analyzed by flow cytometry to the exclusion of propidium iodide-positive dead cells.

### Enumeration of enteric neurospheres

Dissociated enterocytes were cultivated non-adherently in SmartDish™ 6-well plates (STEMCELL Technologies) at the cell dose of 2 × 10^5^ in NSM or SRM as described above. On day 7, neurospheres generated were counted using an inverted phase-contrast microscope. For neurosphere enumeration, STEMgrid™-6 counting grid (STEMCELL Technologies) was attached to the bottom of SmartDish™ 6-well plates. Five zones, of which each contained 4 grids of 2 × 2 mm, were labeled for neurosphere counting. In each 35-mm well, neurospheres growing on 20 selected grids were enumerated. Enteric neurospherogenesis was defined by neurosphere (≥ 50 μm in diameter) number per unit area (mm^2^).

### Cultivation of enteric neurospheres in NSM or SRM

Enteric neurospheres or accutase-dissociated neurosphere cells were grown on coverslips or culture wells coated with human fibronectin (10 μg/ml for 60 min; Sigma Chemical Co, St. Louis, MO) and flooded with NSM or SRM. On indicated days, the growth and development of enteric neurospheres were photographed live, or after Diff-Quik or immunofluorescence staining. Moreover, neurosphere activities or neural networks assembled in vitro were subjected to time-lapse microphotography by an automated optical microscope of Leica TCS SP8X STED.

### Flow cytometric analyses for ENSCs, gliocytes and neurons [25]

Flow cytometry was used to define the neural cell populations generated. Colonies in culture wells were first dissociated by incubating in pre-warmed (37 °C) in 0.025% trypsin for 4 min and then quenched by adding twice the volume of flow buffer (2% fetal bovine serum in PBS). Cell suspension was gently triturated by a microliter pipet to prepare a single cell suspension. Following PBS wash and centrifugation (at 220×*g* for 5 min at 25 °C), cell pellets were resuspended in an appropriate volume for staining.

For intracellular staining, cells were fixed, permeabilized and washed with BD Cytofix/Cytoperm™ Kit (BD Biosciences). Samples were first stained with anti-Tubulin β3 (TUBB3 or TUj1, expressed in neurons) and anti-p75 neurotrophin receptor (p75, expressed in ENSCs) primary antibodies, followed by appropriated secondary antibodies conjugated with fluorescence. Following vigorous wash with washing buffer, cell were finally treated with fluorescence-conjugated primary antibodies against S100 calcium-binding protein B (S100b expressed in gliocytes, C-3, Santa Cruz Biotechnology). After the last wash, cells were acquired by BD FACSCantoTM II and analyzed with BD FACSDiva software.

### Quantification of apoptotic p75^+^ ENSCs in NSM and SRM

Cells (2 × 10^5^ in each well of 6-well plates) were grown in NSM or SRM neurosphere cultures for 3 or 6 days and subjected to apoptosis assay, which detected the loss of membrane asymmetry by annexin V binding to externalized phosphatidylserine during apoptosis. To probe apoptotic p75^+^ ENSCs caused directly by NSM or SRM cell culture rather than membrane damage imposed on cells during cell detachment, we incubated culture cells with annexin V-biotin (BioLegend) and its binding buffer before cell harvest [26] according to the manufacturer’s instructions. Then, adherent cells were dissociated by Accutase, collected, washed and resuspended in culture medium. Samples were further treated with fluorescence-labeled streptavidin and surface-stained with p75 (Clone 25-8, LSBio), followed by fluorescence-conjugated secondary antibody. After final wash and propidium iodide added, cells were analyzed by flow cytometry to the exclusion of propidium iodide-positive necrotic cells.

### Immunofluorescence confocal microscopic imaging of cultivated neurospheres

Enteric neurospheres or their dispersed forms grown on fibronectin-coated coverslips in SRM-filled wells were first treated with BD Cytofix/Cytoperm™ Kit. Then, specimens were incubated with primary antibodies against the target proteins of vimentin (Poly29191, BioLegend), TUBB3 (TUJ1, BioLegend), glial fibrillary acidic protein (GFAP, Poly28400, BioLegend), p75 (Clone 25-8, LSBio), neuronal nitric oxide synthase (nNOS, Cat No: ab5586, Abcam) or choline acetyltransferase (ChAT, Cat No: ab34419, Abcam), followed by fluorescence-conjugated secondary antibodies against the species the primary antibodies were raised in. Fluorescence-conjugated anti-S100b or tyrosine hydroxylase (TH, Clone 2/40/15, BioLegend) were added to specimens after the completion of secondary antibody reaction and washing. Counterstain for the nuclei was performed using diamidino-2-phenylindole (DAPI). After washing, specimens were mounted with Dako fluorescence mounting medium and examined under a Leica confocal microscope.

### 3D culture of enteric neurospheres

Enteric neurospheres were cultivated in a 3D environment embedded in hydrogels, using 3-D Life ToGro Hydrogel kit (G94-1, Cellendes, Germany). RGD-Dextran and CD-Link were prepared according to the manufacturer’s instructions. Enteric neurospheres were suspended in 5 µl SRM and mixed with 18 µl RGD-Dextran. Then, 2 µl CD-Link was added to neurosphere-containing RGD-Dextran to initiate gelation by gently pipetting up and down a few times. During the gel forming process, gel mixture of 2–3 µl was immediately seeded as hydrogel microspheres in the culture dishes and kept stationary for 20 min. Once hydrogels had formed, they were flooded with SRM and kept in the incubator.

### Implantation of neurospheres on gut explants in organotypic culture

Murine colon of 2–3 cm was obtained and opened along its length at the mesenteric side. After povidone-iodine disinfection and saline washes, it was flattened with serosal side down on a microscopic slide in the culture dish and mechanically denuded of mucosa by gentle curettages to expose muscularis. Then, gut explants were washed thoroughly using PBS and rinsed with SRM. Enteric neurospheres were implanted on the denuded muscularis surface of gut explants. After left standing for a few minutes to allow neurosphere adhesion, the dishes were carefully filled with SRM to flood the sample slides and kept at 37 °C in a humidified, 5% CO_2_-containing incubator. Culture medium was replaced every 3–4 days.

### Rectal submucosal transplantation of enteric neurosphere cells [27]

Green fluorescence protein-positive (GFP^+^) neurospheres were generated in NSM mass culture of dispersed enterocytes from FVB/NCrl-Tg(Pgk1-EGFP)01Narl murine fetuses and dissociated by Accutase treatment. GFP was used for donor cell tracking after transplantation. Under anesthesia, the anus of a wildtype FVB/N mouse was disinfected using povidone-iodine solution. Then, the rectal mucosa was prolapsed by 3 stay sutures of 6–0 PDS at 2, 6 and 10 o’clock of anorectal junction. GFP^+^ neurosphere cells were circumferentially injected into rectal submucosa at a dose of 2.5–5.0 × 10^5^ in 50 μl saline using an Ultra-Fine II insulin syringe, 31G short needle (Becton, Dickinson and Company). After transplantation, mice were kept on warm blanket till waking up.

### Histological examination of engraftment by immunofluorescence staining

The recipient’s anorectum (the most distal 1 cm colon) was harvested, fixed in 4% paraformaldehyde overnight and embedded in paraffin. Tissue sections were deparaffinized, rehydrated and then subjected to heat-induced antigen retrieval. After permeabilized with Tween-20 and blocked with 1% bovine serum albumin, the sections were incubated with primary antibodies against GFP (1:200, GeneTex) for 1.5 h, followed by fluorescence-conjugated donkey anti-rabbit IgG (Poly4064, BioLgend), and finally treated with anti-TUBB3 (TUJ1, BioLegend) and anti-GFAP (2E1.E9, BioLegand) directly conjugated with fluorescence. Visualization of the nuclei was achieved by Hoechst 33342 staining (1: 20,000, Invitrogen). Sections were mounted with Dako fluorescence mounting medium. Images were taken using a Leica confocal microscope.

### Statistical analyses

All numerical data were shown in box plots. The equality of means was examined by Student’s *t*-test between two independent groups. Differences were regarded as significant in all tests at *p* < 0.05.

## Result

### Neurospherogenic capacity of NSM and SRM in mass cultures of dispersed enterocytes

**Fig. 1:**
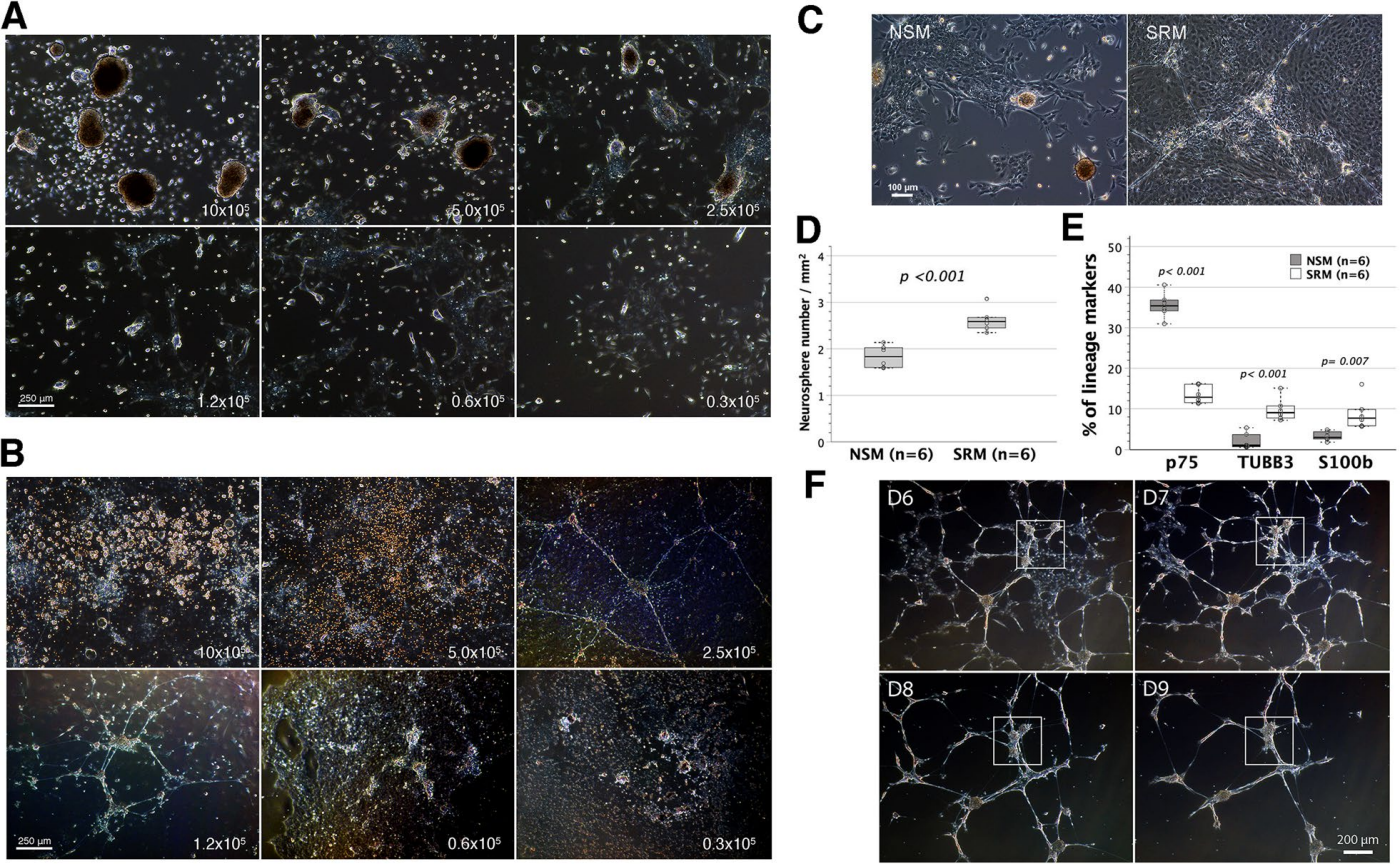
The influences of enterocyte doses on neurospherogenesis in mass cultures of dispersed enterocytes. Mass cultures of dispersed enterocytes (0.3–10 × 10^5^) were carried out non-adherently in 6-well plates flooded with NSM or SRM for ENSC enrichment in the form of neurospheres. On day 7–8, enteric neurospheres were observed and photographed live under a phase-contrast microscope with a 4 × objective. **A** In NSM, neurospheres developed at an enterocyte doses of ≥ 1.2 × 10^5^, but were discernibly smaller in size at the dose of 1.2 × 10^5^. **B** In SRM, neurospheres were recognizable at all the doses used, whereas neuronal wiring did not show up until a dose of 1.2 × 10^5^ cells seeded. At the enterocyte dose of 5–10 × 10^5^, numerous floating cells were present in culture wells. Data shown were a representative set of images from 3 independent experiments using different batches of enterocytes. **C** A representative magnified image at the enterocyte dose of 2.5 × 10^5^ showed the sparsely populated background in NSM by adherent cells, as opposed to the densely populated background in SRM. **D** Neurospherogenesis in equivalent 7-day mass cultures of 2.0 × 10^5^ dispersed enterocytes was quantified in SmartDish™ 6-well plates flooded with NSM or SRM. SRM generated more neurospheres (≥ 50 μm in size) per unit area than NSM (*t*-test). **E** Cells were trypsinized, stained for p75, TUBB3 and S100b, and then analyzed by flow cytometry. SRM compared poorly in p75^+^ but favorably in TUBB3^+^ and S100b^+^ cell fractions with NSM (*t*-test). **F** After trypsinization, neural networks assembled from dispersed enterocytes in SRM were passaged in adherent SRM conditions for twice. Background adherent cells significantly lessened in passaged neural networks. Images were taken live on days 6–9 after the final passage. The neurospheres in the networks were motile. They might fuse together after migration (boxed area)

We first examined the influence of enterocyte doses on ENSC isolation in the form of neurospheres. Dissociated fetal enterocytes were nurtured non-adherently in 6-well plates at escalating doses of 0.3 × 10^5^, 0.6 × 10^5^, 1.2 × 10^5^, 2.5 × 10^5^, 5.0 × 10^5^ and 10 × 10^5^ in NSM and SRM, respectively. NSM gave rise to discrete units of neurospheres, which were discernible under a low-power (4x) objective at a seeding dose of ≥ 1.2 × 10^5^ enterocytes (Fig. 1A). In contrast, neurospheres were identifiable in SRM at all the cell doses used (Fig. 1B). Remarkably, neurospheres in SRM were interconnected by neurite bundles to form neural networks when the seeded enterocytes were at a dose of ≥ 1.2 × 10^5^. However, enterocytes of ≥ 5.0 × 10^5^ overpopulated the culture wells, resulting in numerous floating cells to interfere with network inspections under a microscope. Therefore, the seeding doses of enterocytes in mass culture critically affected the neurospherogenic outcome of NSM and SRM. Enteric neural networks assembled at the dose of 1.2–2.5 × 10^5^ enterocytes in 6-well SRM-flooded plates were optimal for microscopic recordings.

Enteric neural networks assembled in SRM were found to overlie a densely populated background by adherent cells. This contrasted with the situation observed in NSM, where discrete neurospheres were scattering among relatively sparse adherent cells in the background (Fig. 1C). Thus, NSM benefitted neurosphere isolation from enterocytes. We additionally conducted equivalent mass cultures of 2.0 × 10^5^ dispersed enterocytes in SmartDish™ 6-well plates flooded with NSM or SRM to quantify their neurospherogenic capacities. After 1-week cultivation, neurospheres were enumerated under low-power objectives (10x). On average, NSM generated 1.84 neurospheres /mm^2^, whereas SRM gave a yield of 2.62 neurospheres /mm^2^ (Fig. 1D). Statistically, SRM was superior in neurospherogenesis to NSM. ENSC enrichment was further quantified by p75-expressing ENSCs [28–30]. Dispersed enterocytes pooled from a litter of gestational day 14 murine fetuses were measured to contain an average level of 4.65% (3.56–5.86%, *n* = 6 litters) p75^+^ ENSCs. Following mass culture for one week, the mean p75^+^ ENSC fraction increased to 35.6% in NSM and 13.5% in SRM. NSM yielded a significantly higher p75^+^ ENSC fraction than SRM, whereas SRM generated more TUBB3^+^ neurons (9.8% vs 2.1%) and S100b^+^ gliocytes (8.8% vs 3.2%) than NSM (Fig. 1E).

Cell passages in NSM and SRM might lower the cellularity of background adherent cells, leading to the mass production of free-floating neurospheres in NSM (Additional file 1: Movie 1) and better microscopic profiles of enteric neural networks in SRM (Fig. 1F). Enteric neurospheres either in the networks or as isolated units were motile and might fuse with each other in cell culture (Fig. 1F, Additional file 2: Movie 2).

### Quantification of mitotic and apoptotic p75^+^ ENSC fractions in NSM and SRM

**Fig. 2:**
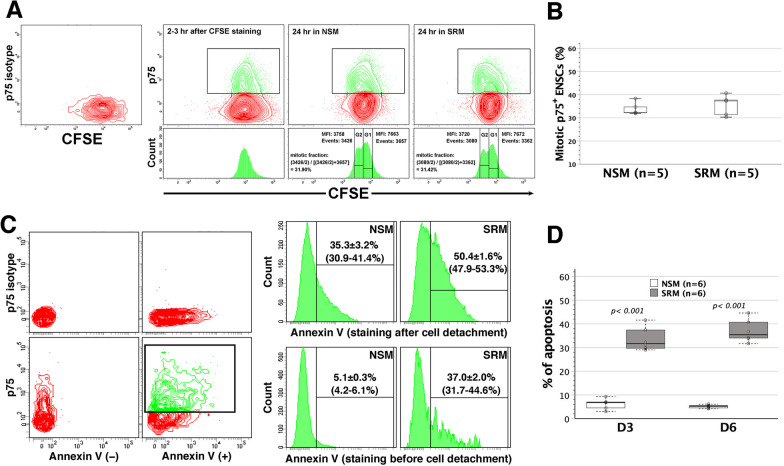
The measurement of mitotic and apoptotic p75^+^ ENSC fractions in NSM and SRM. **A** Dispersed enterocytes were first labeled with CFSE and then grown in NSM and SRM, respectively, for 24 h. Cells were subjected to surface-stained with anti-p75 and Alexa Fluor 647-conjugated secondary antibody, and analyzed by flow cytometry. Defined by isotype controls, the population of p75^+^ ENSCs was gated (green) in contour plots (upper panels), and presented in histogram plots (lower panels). On day 0, p75^+^ ENSCs exhibited a single peak of CFSF fluorescence intensity. After 24-h cultivation in NSM or SRM, a fraction of p75^+^ ENSCs underwent mitosis and led to the second peaks (G2) in histograms, which had the reduction of CFSE mean fluorescence intensity (MFI) by half as compared to the MFI of the original peaks (G1). Representative datasets shown were derived from the same batch of dispersed enterocytes, illustrating the calculation process of mitotic p75^+^ ENSC fractions in NSM and SRM, respectively. **B** Within 24-h cultivation, the mitotic fractions of p75^+^ ENSCs did not differ between NSM and SRM culture conditions (*p* = 0.515). **C** Cells nurtured in NSM or SRM were subjected to apoptosis assays. The system was setup using neurosphere cells stained after cell dissociation by Accutase (left panels). The population of p75^+^ ENSCs was gated (green) and analyzed in histogram plots (right panels). The figures presented were representative histograms of day 6 NSM and SRM-cultivated cells with annexin V staining after (*n* = 3) or before cell detachment (*n* = 6), showing significantly higher fractions of apoptotic p75^+^ ENSCs harvested from NSM (*p* = 0.010) and SRM (*p* = 0.003) cultures in the case of annexin V staining after cell detachment. The fractions shown were mean ± standard error of mean with their ranges in parentheses. **D** Neurosphere cells were stained with annexin V before cell detachment on days 3 (D3) and 6 (D6), respectively, SRM led to a higher fraction of apoptotic p75^+^ ENSCs than NSM at both time points. Apoptosis of p75^+^ ENSCs did not differ between D3 and D6 in NSM (*p* = 0.263) and SRM (*p* = 0.248)

CFSE, covalently and irreversibly coupling to intracellular molecules, is evenly allocated to two daughter cells following cell division. Thus, the proliferating cells can be identified by flow cytometry based upon the reduction of fluorescence intensity by half in daughter cells [24]. In combination with immunophenotyping of p75^+^ ENSCs, CFSE labeling enabled us to not only track proliferating ENSCs within mixed cell populations but also estimate the percentage of ENSC subsets that underwent mitosis. After 24-h culture of dispersed enterocytes in vitro, the mitotic p75^+^ ENSCs could be tracked by their half-reduction of CFSE fluorescence intensity in contour plots and histograms (Fig. 2A). The number of mitotic ENSCs required to generate daughter ENSCs equaled the number of daughter cells divided by 2. Thus, the proportion of mitotic ENSCs could be determined by dividing the number of mitotic ENSCs by the sum of undivided and mitotic ENSC numbers. It was estimated that 31.90–38.33% of p75^+^ ENSCs proliferated in NSM, comparable to 30.30–40.65% of mitotic p75^+^ ENSCs in SRM within 24-h cultivation (Fig. 2B). It indicated that the mitotic capacity of p75^+^ ENSCs was similar in NSM and SRM. Early apoptosis of p75^+^ ENSCs was quantified by annexin V binding to apoptosis-related externalized phosphatidylserine of p75 surface-stained cells in NSM and SRM cultures. There was a significantly higher fraction of apoptotic p75^+^ ENSCs in NSM and SRM cultures in the context of annexin V staining after cell detachment, as compared with staining before cell detachment (Fig. 2C). It indicated that cell detachment exacerbated apoptosis, leading to the overestimation of apoptosis when cells were annexin V-stained after cell detachment. We quantified apoptosis of p75^+^ ENSCs by staining cells with annexin V before cell detachment. SRM gave rise to a higher apoptotic state of p75^+^ ENSCs than NSM in both days 3 and 6 cultures, whereas it made no difference in apoptosis between days 3 and 6 p75^+^ ENSCs in either NSM or SRM (Fig. 2D).

### Characterization of enteric neural networks assembled from dispersed enterocytes

**Fig. 3:**
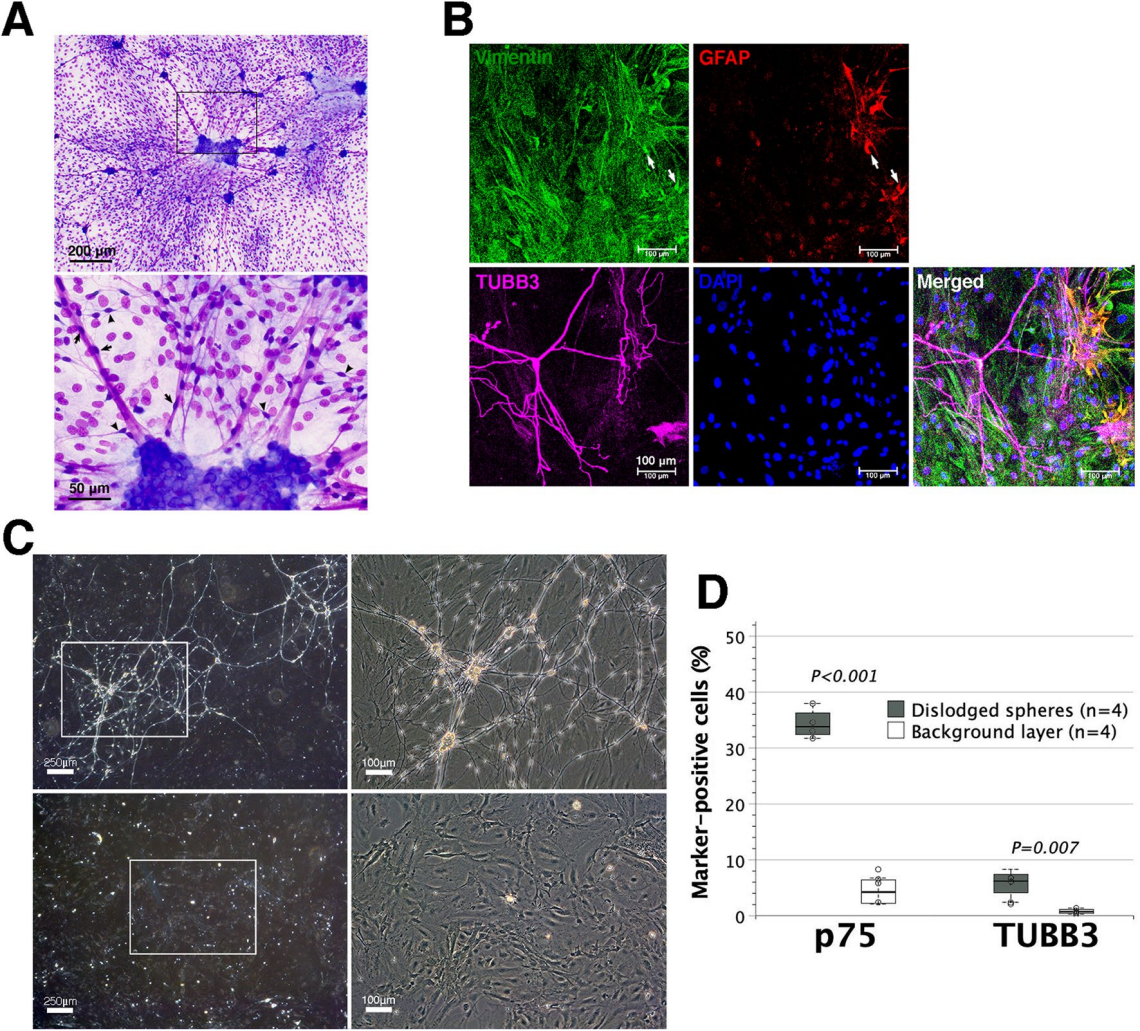
Enteric neural networks assembled from dispersed enterocytes. Fetal enterocytes (2 × 10^5^) were grown non-adherently in SRM for one week to assemble neural networks overlying a layer of adherent cells in the background. **A** On Diff-Quik staining, background cells had round-to-oval and large nuclei, which were stained lighter than those of neural cells. Enteric gliocytes had lanky and bipolar processes, either running with (arrows) or bridging between (arrowheads) interspheric nerve fibers. Neurospheres within enteric neural networks resembled ganglia. **B** Immunofluorescence staining showed TUBB3^+^ neurons and GFAP^+^ gliocytes in neural networks. Background adherent cells were almost positive for vimentin, which is ubiquitously expressed in normal mesenchymal cells. GFAP^+^ cells that were positive for vimentin (arrows) could be developing gliocytes. **C** Neurospheres in the networks were dislodged gently by pipetting from mesenchymal cell layers. Dislodged neurospheres and trypsinized mesenchymal cell layers were, respectively, passaged in fibronectin-coated wells flooded with SRM for additional two weeks. Neural networks could be recovered by dislodged neurospheres (upper panels) rather than adherent mesenchymal cell layers (lower panels). **D** Further flow cytometric analyses disclosed that neurospheres comprised far more p75^+^ ENSCs and TUBB3^+^ enteric neurons than adherent mesenchymal cell layers

On Diff-Quik staining, enteric neural networks assembled in SRM were made up of ganglion-like neurospheres, interspherical nerve bundles and a scattering of elongated gliocytes, either running with or bridging between nerve fibers overlying a background layer of adherent cells (Fig. 3A). Immunofluorescence staining demonstrated that neural networks comprised TUBB3^+^ neurons and GFAP^+^ gliocytes overlying a layer of primarily vimentin-positive mesenchymal cells [31, 32] (Fig. 3B). GFAP^+^ gliocytes stained with vimentin could be immature forms because vimentin was transiently expressed in developing gliocytes [33, 34].

To collect the neurospheres and further examine their ability to recover neural networks, we gently sloshed SRM back and forth by pipetting to dislodge neurospheres. Then, the remaining mesenchymal cells tightly adherent to the bottom were trypsinized. Both were subcultured in SRM-flooded fibronectin-coated wells, respectively. The passage of dislodged neurospheres in SRM restored enteric neural networks with sparse mesenchymal cells in the background, whereas the passage of trypsinized mesenchymal cells hardly recovered neurospheres, let alone neural networks (Fig. 3C). Additional flow cytometric examinations revealed that p75^+^ ENSCs and TUBB3^+^enteric neurons mainly dwelt in ganglion-like neurospheres of enteric neural networks rather than mesenchymal cell background layers (Fig. 3D).

### Neural network assembly of enteric neurospheres

**Fig. 4:**
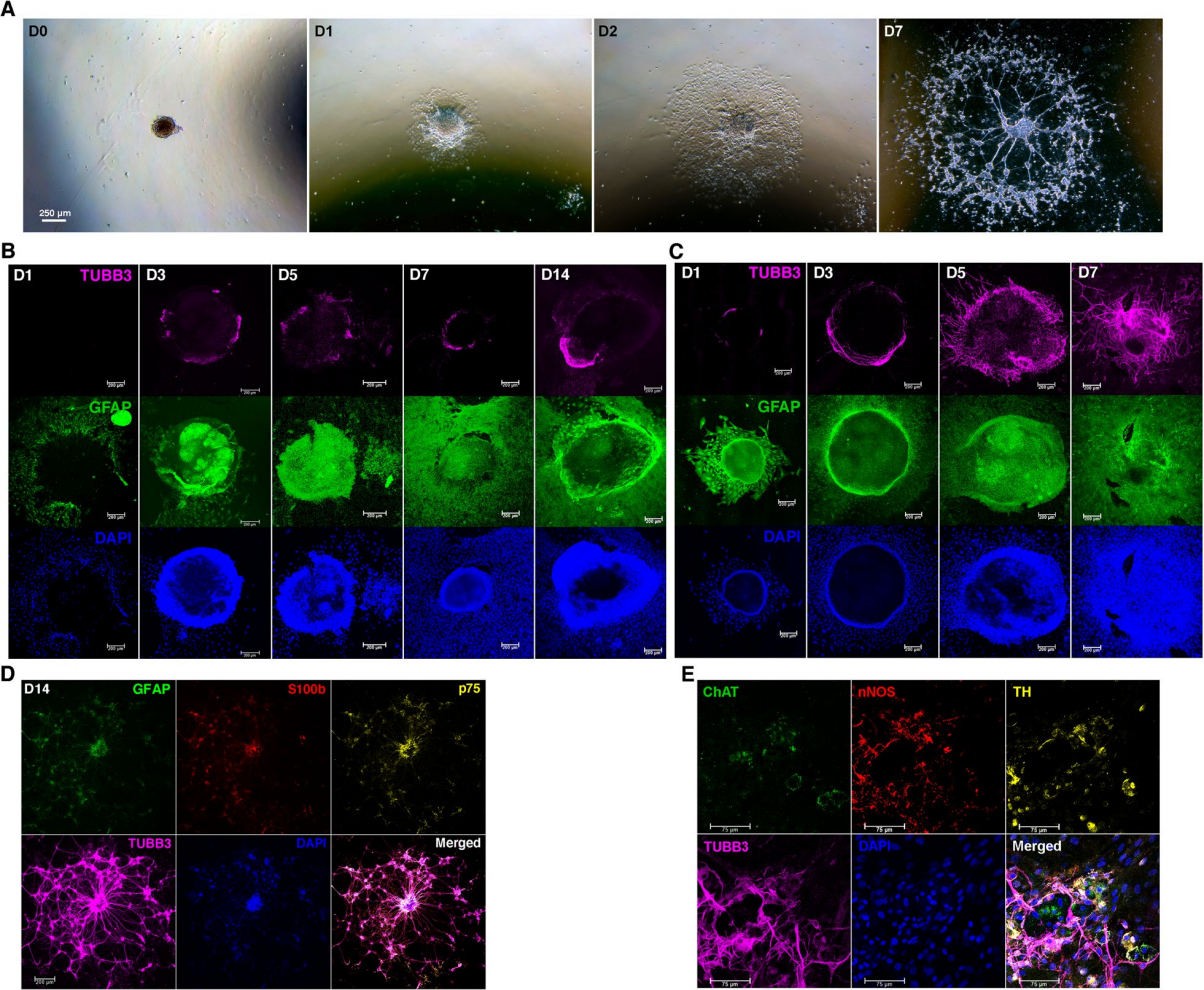
Migration and differentiation of enteric neurosphere cells. Enteric neurospheres generated in NSM were grown on fibronectin-coated coverslips flooded with NSM or SRM. **A** Neurosphere cells migrated radially outward to assemble neural networks. The area populated by migratory neurosphere cells approximated a circle, photographed live on days 0, 1, 2 and 7. (D0, 1, 2 and 7). The representative images were taken in SRM culture. **B** Samples were subjected to immunofluorescence staining. In NSM, the neurospheres hardly stretched out their neurites, nor did they develop any enteric neural network within 2-week cultivation. **C** In SRM, GFAP^+^ gliocytes first migrated out of the spheres after seeding. Neurites began to outgrow on D3. **D** By D14, enteric neurospheres in SRM assembled neural networks with numerous ganglion-like spheres interconnected by neurite bundles. The ganglion-like spheres within the networks consisted of p75^+^ ENSCs, TUBB3^+^ neurons, GFAP^+^ or S100b^+^ gliocytes. **E** SRM might drive ENSCs to differentiate toward a variety of enteric neurons that synthesized cholinergic (ChAT), nitrergic (nNOS) and catecholaminergic (TH) neurotransmitters. Images were taken on D12

Enteric neurospheres generated in NSM were seeded on fibronectin-coated coverslips flooded with SRM. The cell activities of neurospheres could be photographed live or after staining under a phase-contrast or bright-field microscope. Neurosphere cells moved radially outward from the neurospheres (Fig. 4A and Additional file 3: Movie 3). Immunostaining showed that migratory cells were mainly GFAP^+^ gliocytes (Fig. 4B, C). Following outward expansion of enteric gliocytes, there was hardly neuronal differentiation in NSM, let alone neuronal network assembly (Fig. 4B). In contrast, neuronal differentiation with neurite outgrowth was evident on around day 3–5 in SRM (Fig. 4C), followed by neural network assembly in the ensuing days (day 7–14, Fig. 4C, D). Time-lapse microphotography revealed that neurite extension was led by outward expansion of gliocytes (Additional file 4: Movie 4). Neural networks contained numerous neurospheres that harbored p75^+^ ENSCs, TUBB3^+^ neurons, and GFAP^+^ or S100b^+^ gliocytes (Fig. 4D). Thus, neurospheres within neural networks structurally mimicked ENS ganglia. ENSCs in SRM might differentiate toward a broad range of neuronal phenotypes, at least exhibiting cholinergic, nitrergic and catecholaminergic activities (Fig. 4E).

### Neural networks assembled from dissociated neurosphere cells

**Fig. 5:**
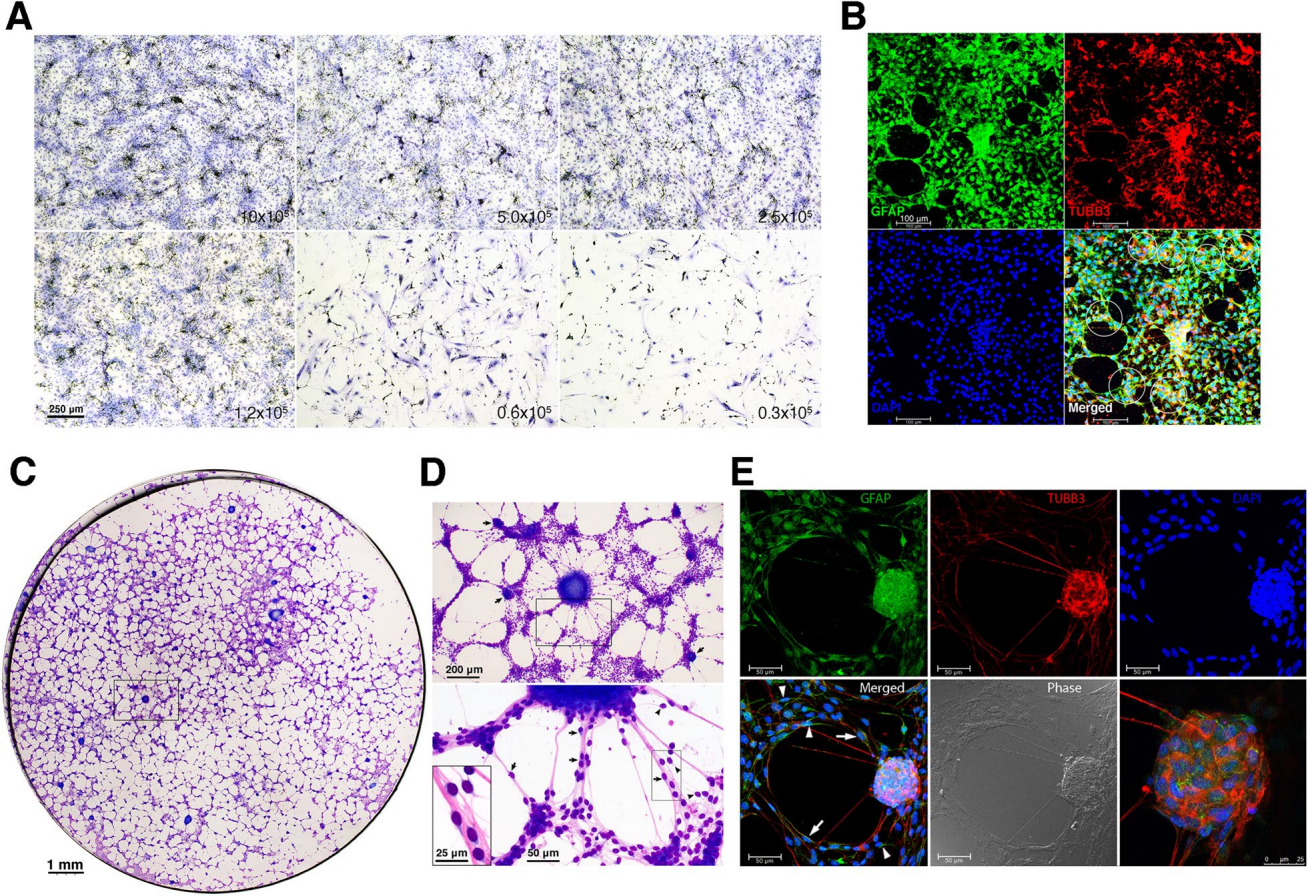
Construction of enteric neural networks in vitro. **A** Dissociated neurosphere cells were nurtured adherently at serially diluted doses of 10–0.3 × 10^5^ in 6-well plates flooded with SRM. Neuronal wiring with network assembly was extensive at the doses of 10–1.2 × 10^5^, but unremarkable at the doses of 0.6–0.3 × 10^5^ with a sparsely populated background by gliocytes. Images were taken on day 7 culture after Diff-Quik staining, and representative of three independent experiments using different batches of enteric neurospheres. **B** The coverslips in wells were subjected to immunofluorescence staining. GFAP^+^ gliocytes densely populated the background. The central ganglion-like neurosphere extended its neurites radially toward neighboring aggregates of neurons and gliocytes (circled area) as developing ganglia. **C** On day 30, the coverslips were subjected to Diff-Quik staining to show the configuration of extensive enteric neural networks. The networks mimicked ganglionated plexuses. It had the ganglion (the number of network nodes as intersections of neurite bundles) density of 20–30 /mm^2^. This panorama was created by image stitching using Photoshop. **D** The magnification of boxed area illustrated a 200 µm spherical ganglion with several neurites radiating toward other surrounding smaller ganglia of < 100 µm (arrows, upper panel). A zoom-in image of boxed area showed lanky gliocytes, which were either closely attached to eosinophilic neurites (arrows, lower panel) or as a bridge between neurites (arrowhead). An inset in the left lower corner showed gliocytes with basophilic cytoplasm. **E** Immunofluorescence staining showed a spherical ganglion with its neurite extension. The sphere, sized 50 µm in diameter, had neurite projections from cell bodies of TUBB3^+^ enteric neurons. GFAP^+^ gliocytes were dwelling in the ganglion, adhering (arrows) to, or bridging (arrowheads) between neurites

We further evaluate whether enzymatically dissociated neurosphere cells could assemble neural networks. Dissociated neurosphere cells were grown adherently at serially diluted doses in 6-well plates flooded with SRM. Neuritogenesis with network development dramatically diminished when the dose of neurosphere cells was less than 1.2 × 10^5^ (Fig. 5A). It suggested that neural network assembly in vitro demanded a threshold density of dissociated neurosphere cells in wells. Likewise, neural networks assembled from dissociated neurosphere cells in SRM were preceded by the proliferation and expansion of GFAP^+^ gliocytes (Fig. 5B).

Prolonged cultivation of dissociated neurosphere cells or multiple neurospheres in SRM for 2–4 weeks might give rise to the assembly of widespread enteric neural networks (Fig. 5C), morphologically similar to ENS ganglionated plexuses. This in vitro neural network had the ganglion density of 20–30 /mm^2^. Outside the ganglia, the gliocytes either adhered to or bridged between the neurite bundles (Fig. 5D, E), comparable to the gliocyte setup within neural networks assembled from dispersed enterocytes (Fig. 3A). Time-lapse microphotography (Additional file 5: Movie 5) revealed that ENS networks were not static but rather dynamic with constant changes of their configurations. There were numerous nerve cells, morphologically similar to gliocytes, moving in and out of the ganglia. The ganglia themselves might slowly transform in shape and migrate along neurite bundles to fuse together. On the neurite bundles of neural networks, enteric gliocytes were also motile, capable of crawling along or off neurites. Moreover, gliocytes did not have a fixed shape, but rather underwent metamorphosis during migration in the forms of bipolarity, tripolarity and even tetrapolarity. There was no wrapping of enteric glial processes around the neural neurites. These results supported the notion that enteric neurospheres committed to the assembly of enteric neural networks and also suggested that ENS might be the dynamic networks with highly motile gliocytes and ganglia.

### Enteric neural networks assembled on gut explants and in 3D environments

**Fig. 6:**
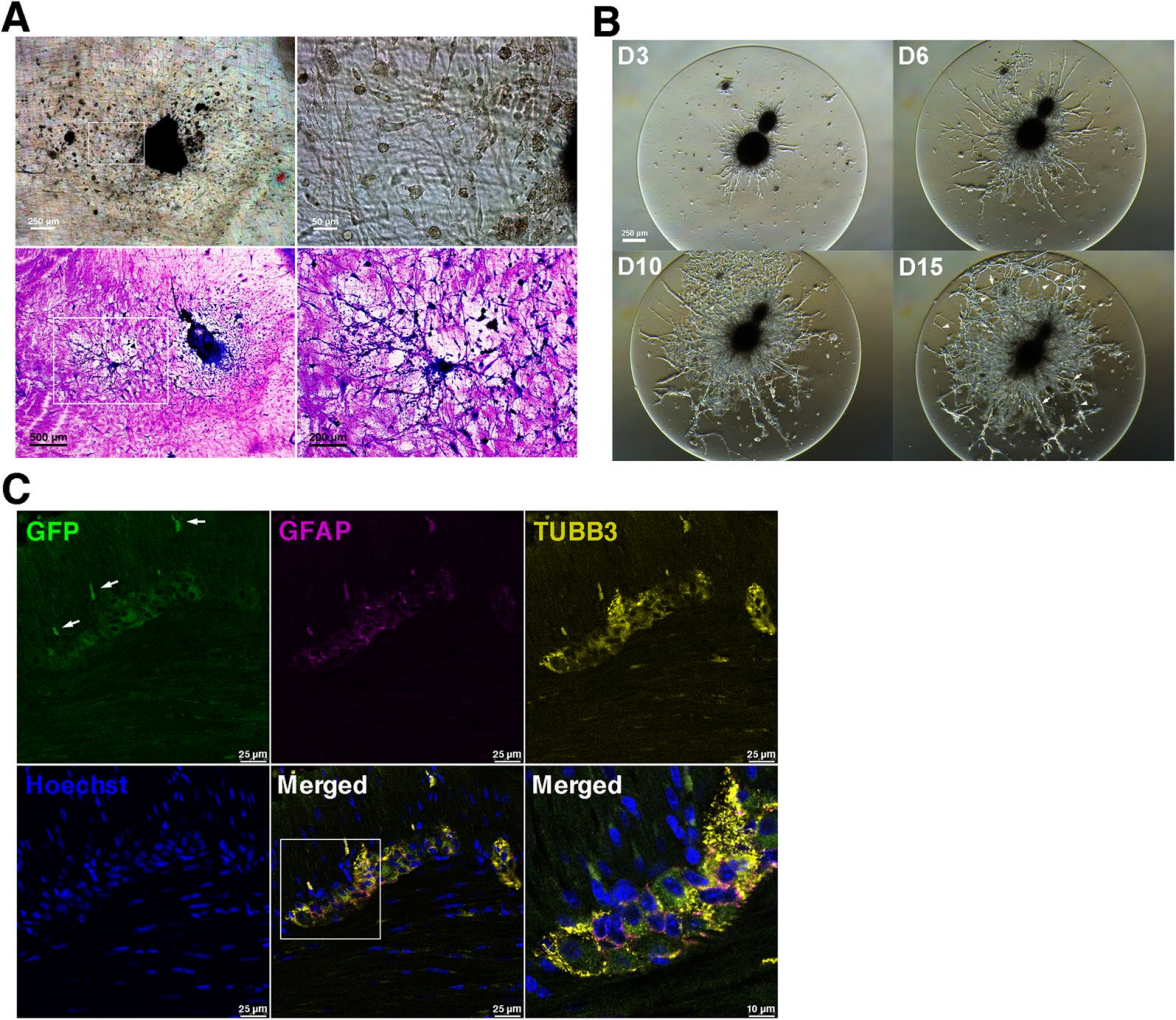
Neural network assembly on gut explants and in 3D hydrogels and reconstitution of colonic myenteric plexuses by donor neurospheres. **A** Neurospheres were implanted on the muscularis of gut explants denuded of mucosa. Neurosphere cells could migrate on the muscularis (upper panels, images taken live on D6) and finally give rise to neural network assembly (lower panels, images taken after Diff-Quik staining on D14). **B** Enteric neurospheres gown in SRM-immersed hydrogels for 2 weeks gave rise to the assembly of 3D neural networks. Arrows and arrowheads pointed to the neurite bundles with different image sharpness in a 3D environment, reflecting the distinct depth of focus under a microscope. **C** GFP^+^ enteric neurospheres dissociated by Accutase were transplanted into rectal submucosa of wildtype recipient mice. Engraftment was examined by immunofluorescence staining of recipient’s anorectum one week after transplantation. Confocal microscopy disclosed that a representative myenteric ganglion contained enteric neurons and gliocytes of donor origin. The neurites outgrowing in the outer muscle layer were of donor origin (arrows). A zoom-in view of donor-origin ganglion (boxed area) was taken after additional scanning of the fluorescence microscope. Images shown were representative of 3 recipients subjected to neurosphere transplantation

Enteric neurospheres were implanted on the denuded muscularis layer of the gut explants maintained in SRM organotypic culture so as to test their capability of migration and network assembly ex vivo. It showed that neurosphere cells could migrate on the muscularis and eventually assemble neural networks on gut explants (Fig. 6A). A 3D hydrogel culture model was used to simulate an extracellular-matrix microenvironment and examine whether isolated neurospheres could assemble ENS networks in 3D environments. This SRM-supplemented 3D culture system created one more dimension for cell adhesion and biochemical or mechanical inputs, and supported the ability of enteric neurospheres to develop the 3D ENS networks (Fig. 6B, Additional file 6: Movie 6).

### Transplantation of enteric neurospheres

To evaluate whether ENSCs or their progenies could reconstitute ENS ganglia in vivo, we performed rectal submucosal injection of dissociated neurosphere cells after trans-anal prolapse of rectal mucosa in a syngeneic model of FVB/NCrl-Tg(Pgk1-EGFP)01Narl into wildtype FVB/N mice. One week later, the recipient’s anorectum (the most distal 1 cm colon) was harvested, fixed and subjected to immunofluorescence staining. Confocal microscopy demonstrated that donor-origin enteric neurons and gliocytes repopulated intermuscular area of the colon and assembled myenteric ganglia with neurite extension in muscularis (Fig. 6C), suggestive of network assembly. Thus, donor ENSCs or their progenies within the mass-produced enteric neurospheres proved a viable source to reconstitute myenteric plexuses of the colon.

## Discussion

Neural stem cells or precursors were first found to exist in neural cell population dissociated from adult murine brain [35]. They could proliferate, self-renew and differentiate toward neurons and astrocytes in cell culture systems [36] to cluster together in a fashion of free-floating spheres, known as neurospheres [37]. ENSCs mainly arise from vagal and sacral neural crest cells that migrate and colonize the gut mesenchyme during embryogenesis to form ENS [38]. The mesh-like ENS is embedded in the gut wall and mixed with a variety of surrounding cells. Thus, ENSC isolation has long since been a technical challenge. It was not until the discovery of surface markers on post-migratory crest-derived cells within the bowel wall along with their antibodies available in early 1990s [39] that researchers were able to immunoselect ENSCs from dispersed enterocytes [18, 19, 40, 41]. However, ENSC surface markers currently available are not regarded as robust enough to prospectively encompass the ENSC population at different stages of development [42]. Moreover, the rarity of ENSCs (< 0.2%) [19] in the gut wall made ENSC immunoselection cost-ineffective and clinically inapplicable, considering the requirement of sacrificing massive autologous bowel to recover sufficient ENSCs. In 2003, Bondurand et al. first described the neurosphere-like bodies generated in cell culture of dispersed enterocytes [3]. They shared the similarities of neurogenic and gliogenic potentials with neurospheres from the brain. Thereafter, the strategy for ENSC enrichment moved toward the cultivation of heterogenous enterocyte populations under specific culture conditions to benefit ENSC survival, proliferation and gathering in the form of neurospheres [3].

In mass culture of dispersed enterocytes, NSM generated less neurospheres than SRM. Of note, enteric mesenchymal cells also proliferated and expanded in the background of culture wells to interfere with neurosphere isolation. Mesenchymal cells in NSM sparsely populated the background, as opposed to a densely populated background in SRM. However, SRM exhibited its superiority of driving ENSC differentiation toward neurons and gliocytes over NSM. Taken together, NSM gave rise to a higher p75^+^ ENSC fraction of the resulting cells than SRM given comparable mitotic capacity of p75^+^ ENSCs in NSM and SRM. Thus, NSM compared favorably in p75^+^ ENSC-containing neurosphere isolation from dispersed enterocytes with SRM despite numerical superiority of neurospheres enjoyed by SRM. As a favorable neural differentiation medium, SRM had a higher ability to induce apoptosis of p75^+^ ENSCs than NSM. The heightened ENSC apoptosis in SRM might have relevance to the sharing of similar biological processes or pathways between apoptosis and differentiation such as alterations in nuclear morphology, cytoskeletal rearrangements/reorganization and phospholipid reorientation of membrane as well as caspase activation despite contrasting cell outcomes [43]. Moreover, coupling of apoptosis and differentiation regulation at the transcriptional level was hardwired into the developmental program [44], providing the fast and direct contrivance to eliminate abnormal cells in case of aberrant cell differentiation as the evolutionarily conserved cell-intrinsic protection from tumorigenesis. Thus, it made sense that SRM promoted ENSC differentiation in company with heightened ENSC apoptosis, signifying apoptosis as an integral component of cell differentiation [43].

Over the years, the size, amount and composition of neurospheres in colony-forming assay have been employed to determine the proliferative, self-renewal and multipotent capacities of a single founding neural stem cell [36]. Our time-lapse microphotography showed that enteric neurospheres in solitude and networks were motile and capable of undergoing fusion with each other, in common with CNS-derived neurospheres [45]. The occurrence of neurosphere fusion in culture negated the clonal entities of enteric neurospheres and undermined the reliability of neurosphere assay to measure the clonality and number of ENSCs. Thus, the development of enteric neurospheres could not accurately reflected ENSC amount, but remained a useful strategy for ENSC isolation and expansion given the rarity of p75^+^ ENSCs and TUBB3^+^enteric neurons outside the neurospheres in culture systems. In fact, enteric neurospheres generated from unsorted enterocytes in NSM were reportedly heterogenous in their makeup including ENSC derivatives and non-neural cell origins such as smooth muscle actin-positive (SMA^+^) myofibroblasts [14], suggesting the involvement of ENSCs and non-neural cell origins in neurospherogenesis. In contrast, p75^+^ ENSCs nurtured in SRM could be neurogenic, gliogenic and myogenic (SMA^+^ myofibroblasts) [18], indicating the additional capacity of SRM to drive p75^+^ ENSC differentiation toward myofibroblasts. Therefore, there was a possibility that the medium employed to nurture ENSCs might influence the diversity of cell types present within neurospheres.

To induce ENSC differentiation, Metzger et al. applied growth factor-free NSM supplemented with fetal calf serum [16, 46] and sometimes ascorbate-2-phosphate [7, 8]. SRM or its equivalent might drive ENSC differentiation after lowering component (chicken embryo extract and bFGF) concentrations [18, 22] and discontinuing IGF-1 [17, 47]. However, these strategies enabled neuronal and glial differentiation in the absence of characteristic ENS network assembly. In this study, the neurospheres generated in SRM were interconnected by neurite bundles, resembling the ganglia within enteric neural networks. It sharply contrasted with the situation in NSM where the neurospheres developed as isolated units barely interconnected with each other by neurites. This clear distinction could be attributed to the inhibitory effects of epidermal growth factor (supplemented in NSM) on neurogenesis, gangliogenesis and neurite outgrowth, thereby precluding ENS neural network assembly [21]. The differentiated enteric neurons in SRM at least included three phenotypes, evidenced by the presence of relevant enzymes to the synthesis of cholinergic, catecholaminergic and nitrergic neurotransmitters. Thus, in preference to NSM, SRM itself even without any component modifications had the capacity to drive neuronal differentiation and ENS network assembly in 2D and 3D environments as well as on ex vivo gut explants. These results explicitly characterized NSM as being ideal for neurosphere enrichment in mass culture of dispersed enterocytes, but SRM as the differentiation medium capable of inducing neuronal differentiation and network assembly. It’s worth mentioning that either neurosphere isolation by NSM or neuronal wiring by SRM was highly influenced by the seeding doses of dispersed enterocytes. Both became inconspicuous once the enterocytes seeded were less than a threshold dose.

Neural networks assembled from dispersed enterocytes in SRM coexisted with a densely populated background by mesenchymal cells. Given that some fastidious stem cells could not survive, grow and develop without the presence of feeder cells as a substratum to condition the environment [48], it might call into question whether network assembly was completely feeder-dependent. However, neural networks could also develop from a single neurosphere on the feeder-free but fibronectin-coated surface, the muscularis of gut explants or in 3D hydrogel environments. Thus, mesenchymal cells were not indispensable to neural network development, but rather simply provided cellular contacts to allow network assembly. The neurosphere cells of < 1.2 × 10^5^ seeded in 6-well plates led to unremarkable neuronal wiring, reflecting the crucial role of neurosphere cell density in network assembly. Thus, ENS network development could be mechanically directed at the relevance of cell interactions. Loose cell arrangements with the poverty of cell interactions in culture wells might preclude network assembly. In might be the reason why there was an absence of neural network assembly in clonal culture of immunoselected ENSCs [18–20] and enzymatically dissociated enterocytes or neurosphere cells [3, 17]. The interactions were reportedly linked to gliocytes that regulated axonal growth and guidance of neurons by direct contact with axons or indirect modulation of the local milieu through secretory factors to influence neuroconnectivity [49]. The crucial role of gliocytes in neuronal wiring could be further strengthened by our findings that neural network development was preceded by outward expansion of GFAP^+^ gliocytes from a single neurosphere, or the formation of background glial cell layers in the culture of dissociated neurosphere cells. Therefore, the formation of a glial histoarchitecture might provide a permissive environment for axon development [50]. ENS networks assembled on fibronectin-coated coverslips morphologically mimicked myenteric plexuses of human or murine colon on whole mount staining [51, 52], and had a higher density of 20–30 ganglia per mm^2^, as compared with 16 ganglia per mm^2^ in the most neuron-rich area of murine colon [52].

Based on the morphology and location within the gut wall, enteric gliocytes were classified into protoplasmic (I), fibrous, (II), mucosal (III) and intramuscular (IV) subtypes [53]. Time-lapse microphotography disclosed that enteric neural networks were a dynamic structure with motile ganglia and gliocytes. The elongated type II or IV gliocytes on nerve fibers could move off the nerve fibers and undergo dramatic shape changes from bipolarity to tripolarity or tetrapolarity. The highly motile and shapeshifting nature of enteric gliocytes undermined confidence in gliocyte classification based upon their morphology and location in snapshot images as it might not suffice to reflect their ability to migrate and transform into another being. The biological significance of motile and shapeshifting gliocytes within ENS awaits further elucidations by more sophisticated live-cell imaging techniques.

## Conclusion

Despite comparable mitotic capacity of ENSCs in NSM and SRM, this study identified NSM as suitable for mass production of enteric neurospheres due to higher p75^+^ ENSC fractions and less mesenchymal cell contamination, whereas SRM, specifically formulated for self-renewal of neural crest stem cells [23], turned out to be the excellent ENSC differentiation medium, capable of driving neuronal and glial differentiation as well as network assembly. Enteric neurospheres retained the capacity to not only assemble neural networks in vitro in 2D/3D environments and ex vivo on gut explants, but also in vivo reconstitute myenteric plexuses of the colon, justifying their employment as cellular inocula to treat enteric neuropathy. The assembled ENS networks exhibited the dynamic entities of their gliocytes and ganglia. They can be used as a neural network model to further explore neuronal and glial activities within ENS after staining or even in live with the assistance of more sophisticated cell labeling and microscopic imaging technologies. Thus, our results were of biological significance in neurogastroenterology, taking one step forward toward ENS demystification.

### Supplementary Information

**Additional file 1**. **Movie 1: Free-floating enteric neurospheres.** Dispersed enterocytes were nurtured non-adherently in NSM. Following 3 passages every 4–5 days, free-floating neurospheres were mass produced.

**Additional file 2**. **Movie 2: Fusion of enteric neurospheres after migration.** Two enteric neurospheres were photographed live every 6 min for 19 h in the culture well flooded with NSM. These successive images were assembled into a video by iMovie. This time-lapse video demonstrated that enteric neurospheres were motile and might fuse with each other.

**Additional file 3**. **Movie 3: Migration of neurosphere cells.** A single neurosphere was grown adherently in SRM. Images were taken live every three minutes for 22 h by an automated microscope with a 10 × objective. All the images were then assembled into a time-lapse video. It showed the radially outward migration of neurosphere cells.

**Additional file 4**. **Movie 4: Neurite outgrowth of neurospheres.** A single neurosphere was grown adherently in SRM. An automated microscope with a 10 × objective acquired a grid of 15% overlapping live images every three minutes for 24 h. All the images were stitched together into a segmented panorama of cell migration and neurite extension. All the composite images were assembled into a time-lapse video by iMovie. It showed that gliocytes migrated radially outward to lead the neurite outgrowth (open arrows).

**Additional file 5**. **Movie 5: Dynamic change of enteric neural networks.** Dissociated neurosphere cells were nurtured adherently in SRM. On day 18, The dynamics of enteric neural networks was recorded by time-lapse microphotography using an automated microscope to acquire a grid of 15% overlapping live images every five minutes for 48 h. Images at each round of scans were stitched together to create a stitched panorama of neural networks. All the panoramic images were then assembled into a time-lapse video by iMovie. It showed that enteric gliocytes were highly motile, crawling along or off the neurite bundles. During migration, the elongated gliocytes constantly underwent shifts in the shapes of bipolarity, tripolarity (open arrows) and even tetrapolarity (solid arrows). The network ganglia were also motile and might slowly transform in shape along with gliocytes moving in and out of the ganglia. Two neighboring ganglia migrated along the neurite bundle to fuse together.

**Additional file 6**. **Movie 6: Enteric neural networks assembled in 3D hydrogels.** Enteric neurospheres were nurtured in 3D hydrogels flooded with SRM. On day 5, neurospheres were interconnected by outgrowing neurites. This video clip was recorded under a microscope while the focus was being finely adjusted. The image sharpness of different neurite bundles and neurospheres in this developing neural network varied greatly along with the adjustment of the focal depths, reflecting the distinct depths of field (open and solid arrows). It indicated that the assembled neural network was a 3D organoid.

## Data Availability

All data generated or analyzed during this study are included in this article.

## Abbreviations

bFGF: Basic fibroblast growth factor
CFSE: Carboxyfluorescein diacetate succinimidyl ester
ChAT: Choline acetyltransferase
DMEM/F12: Dulbecco's Modified Eagle Medium/Nutrient Mixture F-12
DAPI: Diamidino-2-phenylindole
ENS, CNS and PNS: Enteric, central and peripheral nervous system
ENSCs: Enteric neural stem cells
EGF: Epidermal growth factor
GFAP: Glial fibrillary acidic protein
GFP: Green fluorescence protein
IGF-1: Insulin-like growth factor 1
nNOS: Neuronal nitric oxide synthase
NSM: Neurosphere medium
p75: P75 neurotrophin receptor
PBS: Phosphate-buffered saline
SRM: Self-renewal medium
S100b: S100 calcium-binding protein B
3D: Three-dimensional
TUBB3: Tubulin β3
TH: Tyrosine hydroxylase
